# Systematic Analysis of Sequences and Expression Patterns of Drought-Responsive Members of the HD-Zip Gene Family in Maize

**DOI:** 10.1371/journal.pone.0028488

**Published:** 2011-12-02

**Authors:** Yang Zhao, Yuqiong Zhou, Haiyang Jiang, Xiaoyu Li, Defang Gan, Xiaojian Peng, Suwen Zhu, Beijiu Cheng

**Affiliations:** Key Laboratory of Crop Biology of Anhui Province, Anhui Agricultural University, Hefei, China; Université Paris-Sud, France

## Abstract

**Background:**

Members of the homeodomain-leucine zipper (HD-Zip) gene family encode transcription factors that are unique to plants and have diverse functions in plant growth and development such as various stress responses, organ formation and vascular development. Although systematic characterization of this family has been carried out in *Arabidopsis* and rice, little is known about HD-Zip genes in maize (*Zea mays* L.).

**Methods and Findings:**

In this study, we described the identification and structural characterization of HD-Zip genes in the maize genome. A complete set of 55 HD-Zip genes (*Zmhdz1*-*55*) were identified in the maize genome using Blast search tools and categorized into four classes (HD-Zip I-IV) based on phylogeny. Chromosomal location of these genes revealed that they are distributed unevenly across all 10 chromosomes. Segmental duplication contributed largely to the expansion of the maize HD-ZIP gene family, while tandem duplication was only responsible for the amplification of the HD-Zip II genes. Furthermore, most of the maize HD-Zip I genes were found to contain an overabundance of stress-related *cis*-elements in their promoter sequences. The expression levels of the 17 HD-Zip I genes under drought stress were also investigated by quantitative real-time PCR (qRT-PCR). All of the 17 maize HD-ZIP I genes were found to be regulated by drought stress, and the duplicated genes within a sister pair exhibited the similar expression patterns, suggesting their conserved functions during the process of evolution.

**Conclusions:**

Our results reveal a comprehensive overview of the maize HD-Zip gene family and provide the first step towards the selection of *Zmhdz* genes for cloning and functional research to uncover their roles in maize growth and development.

## Introduction

The homeobox (HB) gene encodes a highly conserved 60–61 amino acid homeodomain (HD), which confers sequence-specific DNA binding function by folding into a characteristic three α-helix structure [1], [2]. The HB gene was first identified in a set of *Drosophila* genes controlling development and has been found in all investigated eukaryotes [3]. Since the discovery of Knotted-1 in maize [4], many plant HD proteins have been isolated and categorized into several different groups based on the presence of conserved domains [2]. HD-Zip protein, one group of these proteins, has only been identified in plants thus far. The characteristic of this group is the presence of a HD and an adjacent leucine zipper (Zip) acting as a dimerization motif [5], [6].

In *Arabidopsis*, 48 out of 110 HB genes have been identified as HD-Zip proteins [7]–[11]. Based on their structures, additional specific domains and functions, HD-Zip genes are further categorized into four classes: HD-Zip I-IV [2], [12]. Members of class I and II share the conserved HD and Zip domains and recognize similar pseudopalindromic binding sites: CAAT-N-ATTG [13]–[15]. Furthermore, HD-Zip II members possess an additional domain, known as CPSCE (based on five conserved amino acids: Cys, Pro, Ser, Cys and Glu), which is closely linked to the downstream of Zip domain and mainly responsible for redox cell state perception [16], [17]. Besides the HD and Zip domains, HD-Zip III and IV genes encode two additional common domains: a START domain with putative function in sterol binding followed by an adjacent region named SAD domain with unknown function [2], [18]–[19]. HD-Zip III proteins have a characteristic MEKHLA domain in the C-terminus, which shares significant similarity with the PAS domain and was proposed to function as a sensory domain involved in light, oxygen and redox signaling. Recent studies have indicated that the MEKHLA domain function as a negative regulator of *Arabidopsis* HD-ZIP III *REV* activity by inhibiting dimerization [2], [20]–[21].

HD-Zip encoding genes have been isolated from various plants, such as *Arabidopsis* [22], rice [13], tomato [23], cotton [24], maize [25] and *Physcomitrella patens* [26]. HD-Zip proteins have been demonstrated to participate in transcriptional regulation of a series of biological processes related to plant growth and development. HD-Zip I genes mainly participate in the regulation of abiotic stress responses, e.g. drought, extreme temperatures, osmotic and light stress [27]–[29]. Expression patterns of the *ATHB5*, -*6*, -*7*, and -*12* are either up- or down-regulated by drought stress or by externally abscisic acid (ABA), indicating that these genes may play a crucial role in regulating plant responses to abiotic stress in an ABA-dependent pathway [22], [30]–[32]. *Oshox4*, a HD-Zip I gene from rice, is believed to be involved in the regulation of stem elongation and other developmental processes. Over-expression of *Oshox4* leads to enhanced tolerance to drought stress [33]. Previous studies have also revealed that other members of this class, such as *ATHB16* and -*52*, are involved in light perception signaling [27], [34]. HD-Zip II genes are mainly regulated by illumination conditions and auxin responses [35], [36]. As a negative regulator, *ATHB2* can recognize its own promoter region and participate in regulating the *Arabidopsis* shade avoidance response [37]. Sunflower *HAHB10*, a HD-Zip II gene, which shares a similar gene structure and expression pattern with *ATHB2*, mainly regulates the plant response to light quality and quantity related to plant development [29]. Recently, two cotton HD-Zip II genes, *GhHB2* and -*4*, were found to play essential roles in the phytohormone signaling pathway that regulates early seedling development, and expression of *GhHB2* and -*4* were shown to be up-regulated in hypocotyls, cotyledons and roots by external GA_3_ treatments [38].

The *Arabidopsis* genome contains five HD-Zip III genes (*REV*, *PHB*, *PHV*, *CAN* and *ATHB8*) [10]. Accumulating evidences have revealed that these genes play significant roles in different developmental processes. *ATHB8*, an *Arabidopsis* HD-Zip III gene, is a positive regulator of vascular cell differentiation, and ectopic expression of *ATHB8* in *Arabidopsis* can increase the production of xylem tissue [39]. HD-Zip IV genes are generally involved in determining outer cell fates [40], [41]. *OCL4* encoding a maize HD-Zip IV gene is expressed in the epidermis of the leaf blades. Functional studies showed that trichome development is inhibited in *Arabidopsis* transgenic plants over-expressing *OCL4*, but ectopic trichomes differentiation has also been observed on the edge of the leaf in *OCL4* RNAi transgenic plants [25].

Maize is an important cereal crop and has become a model plant for the study of genetics, evolution and other basic biological research. The availability of the maize genome sequences has provided an excellent opportunity for whole-genome annotation, classification and comparative genomics research [42]. Although HD-Zip genes have been extensively characterized in *Arabidopsis*, rice and other species [8], [33], [43], a systemic analysis of the HD-Zip gene family in maize, especially for the potential stress-responsive members, has not been reported in such an important species. In this study, 55 putative HD-Zip genes were identified and characterized in the maize genome. Furthermore, we investigated the transcript levels of HD-Zip I genes in response to drought stress which severely affects maize yields. The results provide a biological reference for future studies on the functions of the HD-Zip genes and will be helpful for breeding drought-resistant maize.

## Results

### Identification and annotation of HD-Zip genes in maize

The consensus protein sequences of the HD Hidden Markov Model (HMM) profile were employed as a query to search against the maize genome database with BlastP program. Through this approach, a total of 99 HD-containing sequences were identified. To confirm putative HD-Zip genes in the maize genome, the amino acid sequences of all the 99 proteins were searched for the presence of both HD and Zip domains by Pfam and SMART. As results of an extensive search for HD-Zip genes, 55 non-redundant HD-Zip genes (named *Zmhdz1* to *Zmhdz55*) were confirmed from the original data. The total number of HD-Zip genes identified in maize (55) is a little greater than that in either *Arabidopsis* (48) or rice (48) (Table 1) [8]–[11], [44]. We aligned all the *Zmhdz* genes with *Arabidopsis* and rice HD-Zip genes to generate a phylogenetic tree for classification of maize HD-Zip genes. Based on their relationships with *Arabidopsis* and rice HD-Zip genes, the 55 *Zmhdz* genes were divided into four distinct classes, including class I (17 genes), class II (18 genes), class III (5 genes) and class IV (15 genes) (Table 1).

**Table 1:** Numbers of HD-Zip genes in the maize, rice and *Arabidopsis* genomes.

Category	Maize	*Arabidopsis*	Rice
Class I	17	17	14
Class II	18	10	13
Class III	5	5	9
Class IV	15	16	12
Total number	55	48	48

Although all of the HD-Zip genes encode the conserved HD and Zip domains, their sequences elsewhere are highly diverse. The HD-Zip III and IV proteins are in general longer (600–950aa) than those of the HD-Zip I and II proteins (200–400aa). Furthermore, the molecular weights of these deduced *Zmhdz* proteins have a range from 23.8 kDa to 103.56 kDa. Similar to that reported in *Arabidopsis* and rice [2], [33], the *Zmhdz* genes stand out from a large number of other plant gene families in their bimodal distribution of exons. Class I and II *Zmhdz* genes are generally found to have two to four exons, whereas class III and IV contain multiple exons, ranging from seven to eighteen (Table S1).

### Phylogenetic and structural analysis of *Zmhdz* genes

**Figure 1:**
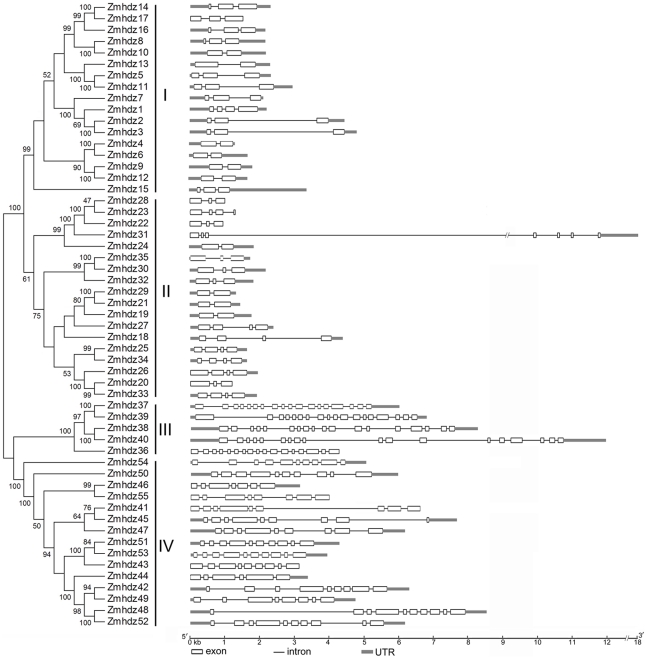
Phylogenetic relationship and gene structure of the 55 predicted maize HD-Zip proteins. NJ tree (the part of left side): The unrooted tree was generated with MEGA4.0 program using the full-length amino acid sequences of the 55 maize HD-Zip proteins. The bootstrap values are indicated at the branches in black numbers, and the proteins were named according to their gene codes (see Table S1). Gene structure (the part of right side): Exons and introns are indicated by white boxes and single lines, respectively. Thick gray lines represent the untranslated regions (UTRs). The length of each HD-Zip gene can be estimated using the scale at the bottom.

The predicted protein sequences of all the *Zmhdz* genes were used to generate an unrooted phylogenetic tree (Figure 1). The unrooted tree categorized the *Zmhdz* genes into four major groups (class I, II, III and IV) with well-supported bootstrap values. Fifty-five of the *Zmhdz* genes formed 18 gene pairs, and all of them had strong bootstrap support (>94%), with the exception of *Zmhdz28*/*23*, *Zmhdz41*/*45* and *Zmhdz51*/*53*. We subsequently performed an exon-intron structure analysis to support the phylogeny reconstruction. The schematic structures revealed that each coding sequence of *Zmhdz* gene is disrupted by one or more introns. Based on those results, the *Zmhdz* genes can be divided into four groups as shown in figure 1. The HD-Zip genes within the same groups of the phylogenetic tree all showed similar exon-intron structures. Similar to that reported in *Arabidopsis* and rice, the numbers of introns are quite different across the four classes. Class III HD-Zip genes contain the most number of introns, while class I and II genes contain the fewest of introns.

**Figure 2:**
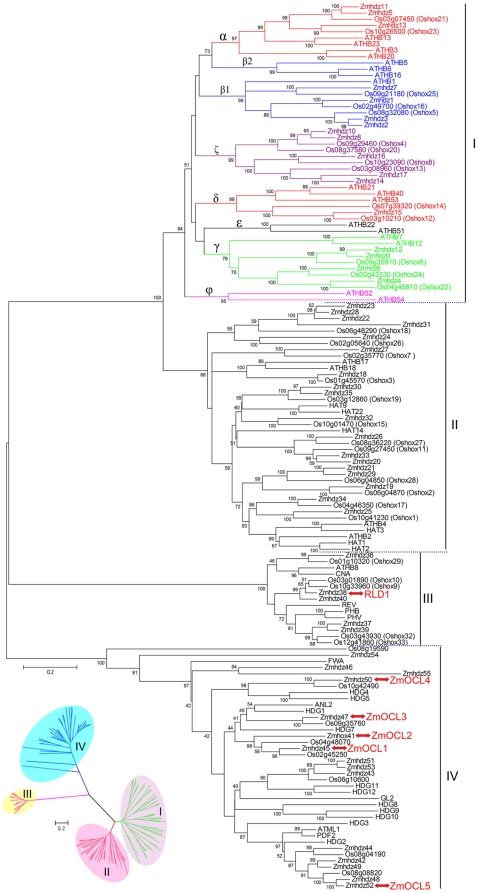
Phylogenetic relationships of maize, rice and *Arabidopsis* HD-Zip proteins. The tree was generated with MEGA4.0 program using the NJ method. Bootstrap values are indicated at the branches in black numbers. The unrooted tree in the lower-left corner was constructed using the same method.

To examine the phylogenetic relationships of HD-Zip genes from different species, a combined phylogenetic tree was constructed from alignment of the full-length sequences of maize, rice and *Arabidopsis* HD-Zip proteins (Text S1). Our results showed that the *Arabidopsis* 48 HD-Zip genes fell broadly into four distinct classes, which contained representative maize and rice HD-Zip genes (Figure 2). In *Arabidopsis*, the HD-Zip I genes have been further divided into six monophyletic subclasses, including α, β, γ, δ, ε and ϕ [8]. In this study, all of the maize and rice HD-Zip I genes can be grouped together with their *Arabidopsis* counterparts except for one clade, which contained nine members of *Zmhdz10*, -*8*, -*16*, -*17*, -*14* and *Oshox4*, -*20*, -*8* and -*13* with high bootstrap support (>95%). Thus, we assigned these nine sequences to the now maize and rice specific ζ clade according to the nomenclature previously proposed by Agalou et al. (2008) [33]. Furthermore, *Arabidopsis* ε and ϕ clades did not group with maize or rice HD-Zip genes. As previously reported in rice, the original β clade was also separated into two clades in our NJ tree, β1 and β2 clades. Clade β1 comprised of four maize and three rice homologs whereas clade β2 did not contain any maize or rice HD-Zip genes. The phylogenetic relationships based on the MP or ML trees were largely consistent with those results (Figure S1 and S2).

The clade of class II genes could be further divided into several subclasses as described by Ciarbelli et al. (2008) [9]. However, a novel subclass, including *Zmhdz23*, -*28*, -*22*, -*31* -*24* and *Oshox18* and -*26*, was shown to evolve separately from the *Arabidopsis* homologs. The NJ tree also showed that *Zmhdz18* and *Oshox3* were closely related to *ATHB17* and -*18*. Although some maize or rice HD-Zip genes could be grouped with their *Arabidopsis* counterparts, the bootstrap values in the tree nodes were low. For example, the bootstrap value of the *HAT14* containing clade was only 51%. In addition, *Zmhdz27* and *Oshox7* formed a sister pair and existed as a single clade.

In plants, class III HD-Zip genes are very conserved, not only in the sequences of HD, Zip and START domains but also in the number. Although class III consisted of just 15 HD-Zip genes from *Arabidopsis*, rice and maize, six sister pairs were found in the NJ tree. Two sister pairs (*Zmhdz37*/*39* and *Oshox32*/*33*) showed close relationship with the *PHB* and *PHV* sister pair. Unlike HD-Zip I and III genes, the phylogenetic relationships of class IV genes were unclear and the bootstrap values were low in some branches. This phenomenon was more significant in the outer lineage-expansion clades, such as the *ANL2* containing clade. Moreover, some independently evolved singletons were also found in this group. Remarkably, *Zmhdz50* and Os10g42490 formed a sister pair and closely related to the *HDG4* and -*5*.

Representatives of the six published HD-Zip genes of class III and IV were also confirmed in the tree. As shown in figure 2, no *Arabidopsis* representatives were grouped with the *Zmhdz38* (*RLD1*) containing clade. In contrary to *RLD1*, *ZmOCL4* showed a close relationship with *HDG4* and -*5*, and this observation was also strengthened by the fact that *ZmOCL4* and *HDG4* and -*5* showed strong expression in flower organs [11]. It should be noted that *ZmOCL1* and -*2* clustered in the same clade, suggesting that genes of this clade may play important roles in outer cell layer development, such as root and floral meristems [45]–[47].

### Investigation conserved motifs in *Zmhdz* genes

**Figure 3:**
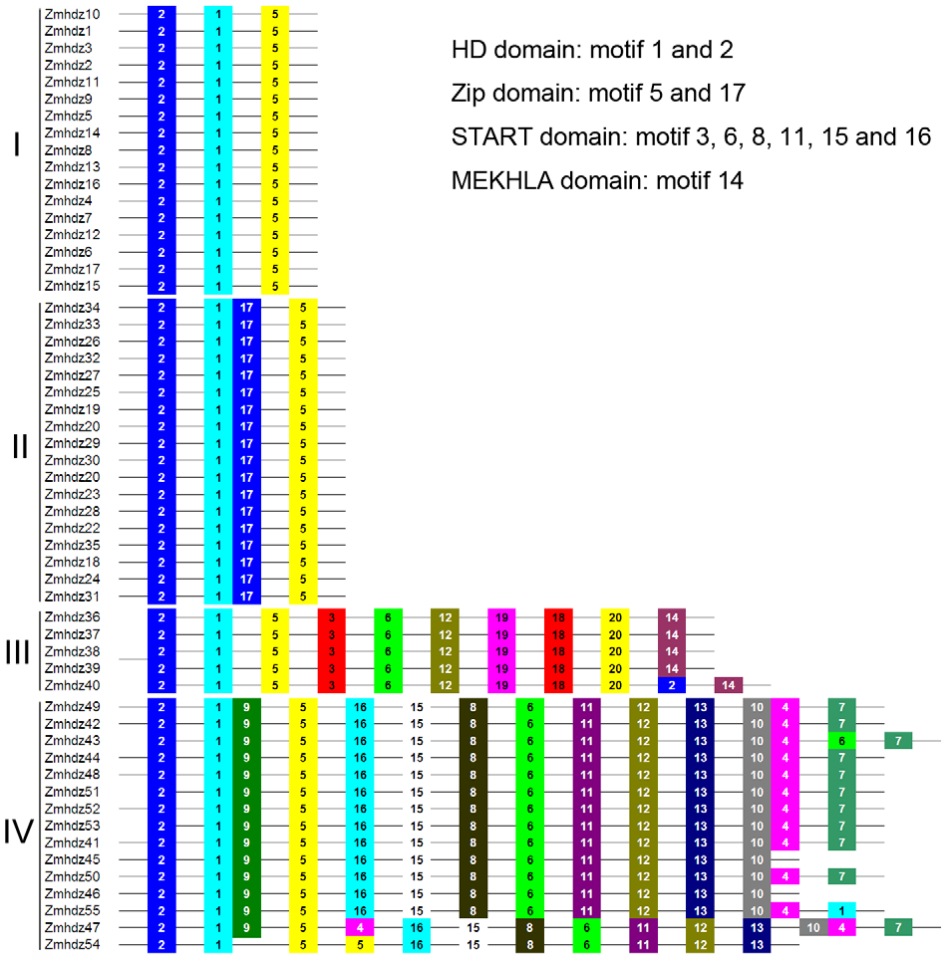
Distribution of 20 putative conserved motifs in maize HD-Zip proteins. Motifs of HD-Zip proteins were identified by MEME web server. Note that the length of each box in the proteins does not represent the actual motif size, and the colored boxes were ordered manually according to the results of the MEME analysis. The conserved amino acid sequences and length of each motif are shown in table 2.

Using the MEME web server, 20 conserved motifs were identified in the maize HD-Zip proteins (Figure 3). Each of the putative motifs obtained from MEME was annotated by searching Pfam and SMART. Based on the distribution of the 20 predicted motifs, we categorized the 55 *Zmhdz* genes into four classes, completely consistent with the classifications from the phylogenetic analysis. Motifs 1 and 2 encoding the HD domain were found in all the 55 *Zmhdz* genes. All of the 55 *Zmhdz* genes had either motif 5 or 17 encoding the Zip domain. The conserved motifs 3, 6, 8, 11, 15 and 16 specifying the START domain were identified in class III and IV genes. The MEKHLA domain, represented by motif 14, was found to be distributed in the C-terminus of each class III gene. In addition, some subfamily-specific motifs were also found with unknown functions, indicating that these motifs are likely required for subfamily-specific functions. The conserved domains and highly conserved amino acid residues encoding subfamily-specific motifs were also confirmed from alignment of the protein sequences of the four classes (Figure S3). The detailed information on conserved amino acid sequences and lengths of the 20 motifs are shown in table 2.

**Table 2:** Major MEME motif sequences in maize HD-Zip proteins.

Motif no.^a^	Width	Conserved amino acid sequences
1	29	LARQLGLQPRQVKVWFQNRRARWKTKQTE
2	21	KKYHRHTKEQIQFLEDCFKEC
3	113	ETLTEFMSKATGTAIDWVQMPGMKPGPDSVGIIAISHGCRGVAARACGLVNLEPTKVIEILKDRPSWYRDCRSMEVYHVIPTGNGGTIELIYMQMYAPTTLAPPRDFWTLRYT
5	15	LTEENDRLQKEIDEL
6	21	PSGCLIQDMPNGYCKVTWVEH
8	37	STGVAGNYNGALQLMYMEFQVPSPLVPTRECYFLRYC
11	23	SGLAFGAHRWVATLQRQCEYLAI
14	76	FANQAGFDMLETTLVNIQDLTLEKIFDEQGRKALYAEIPKIMEQGYAYLPGGVCMSGMGRHVSYEQAVAWKVLGDD
15	29	SGVVIMTHVSLVEIFMDVNKWMEMFPCIV
16	29	DKPMIVELAVAAMDELIRMAQMDEPLWIP
17	11	VDCEYLKRCCE

a Motifs with unknown functions are not shown in this table. The motif numbers are consistent with the numbers in figure 3.

### Chromosomal locations and gene duplication

**Figure 4:**
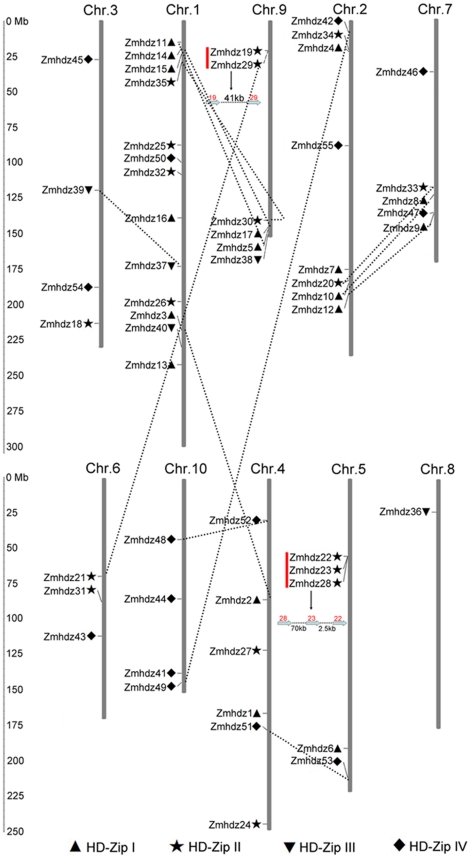
Chromosomal locations of maize HD-Zip genes on all 10 chromosomes. Markers next to the gene names represent the classes to which each HD-Zip gene belongs (HD-Zip I, ▴; HD-Zip II, ★; HD-Zip III, ▾; HD-Zip IV, 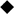). Chromosome numbers are shown at the top of each vertical gray bar, and the names on the left side of each chromosome correspond to the approximate locations of each HD-Zip gene. Genes involved in segmental duplication are joined by dashed lines, and the red rectangle indicates the gene cluster on each chromosome. The scale is in megabases (Mb).

The 55 HD-Zip genes were found to be distributed unevenly across all the ten chromosomes in the maize genome (Figure 4). The number of *Zmhdz* genes appearing on each chromosome had a wide range. Chromosome 1 contains the maximum number of genes (thirteen), followed by chromosome 2 (eight). By contrast, chromosome 8 has only one HD-Zip gene. Relatively high densities of *Zmhdz* genes were found in some chromosomal regions, such as the top of chromosome 1 and the bottom of chromosome 9. In addition, two gene clusters including five genes (*Zmhdz22*/*23*/*28* and *Zmhdz19*/*29*) were detected on chromosomes 5 and 9, respectively.

In this study, gene duplication events, including tandem and segmental duplications, were investigated with the purpose of elucidating the expanded mechanism of the *Zmhdz* gene family that are thought to have occurred during the process of evolution [48], [49]. Based on the phylogenetic analysis and the chromosomal distribution of the *Zmhdz* genes, 12 gene pairs (*Zmhdz14*/*17*, *Zmhdz8*/*10*, *Zmhdz5*/*11*, *Zmhdz2*/*3*, *Zmhdz9*/*12*, *Zmhdz35/30*, *Zmhdz29*/*21*, *Zmhdz20*/*33*, *Zmhdz37*/*39*, *Zmhdz51*/*53*, *Zmhdz42*/*49* and *Zmhdz48*/*52*) were identified to be involved in the segmental duplication events (Figure 4). Among the 12 segmental duplication events, the high frequency of segmental duplication occurred between chromosomes 1 and 9, which contained three segmental duplication events, as well as between chromosomes 7 and 2. According to the B73 maize genome annotation, the clustered genes of *Zmhdz19* and -*29* were spaced by a 41 kb genomic distance and separated by only one gene on the chromosome. The locations of *Zmhdz28*, *22* and *23* were adjacent to each other on the chromosome. Since the fact that genes in the same cluster have high sequence similarity and share close evolutionary traits, all the five genes involved in the two clusters were considered as tandem duplicated genes.

It is noteworthy that among the 18 sister pairs, five gene pairs (*Zmhdz4*/*6*, *Zmhdz25*/*34*, *Zmhdz38*/*40*, *Zmhdz46*/*55* and *Zmhdz41*/*45*) (*Zmhdz28* and -*23* were found to be involved in tandem duplication) were located just outside the segmental duplication regions [50]. However, their gene structure and phylogenetic relationships indicated that they share a similar evolutionary history and are closely related to each other (Figure 1). Therefore, the five gene pairs were determined as putative segmental duplications.

### Identification of putative stress-related *cis*-elements in the promoters of *Zmhdz* I genes

**Figure 5:**
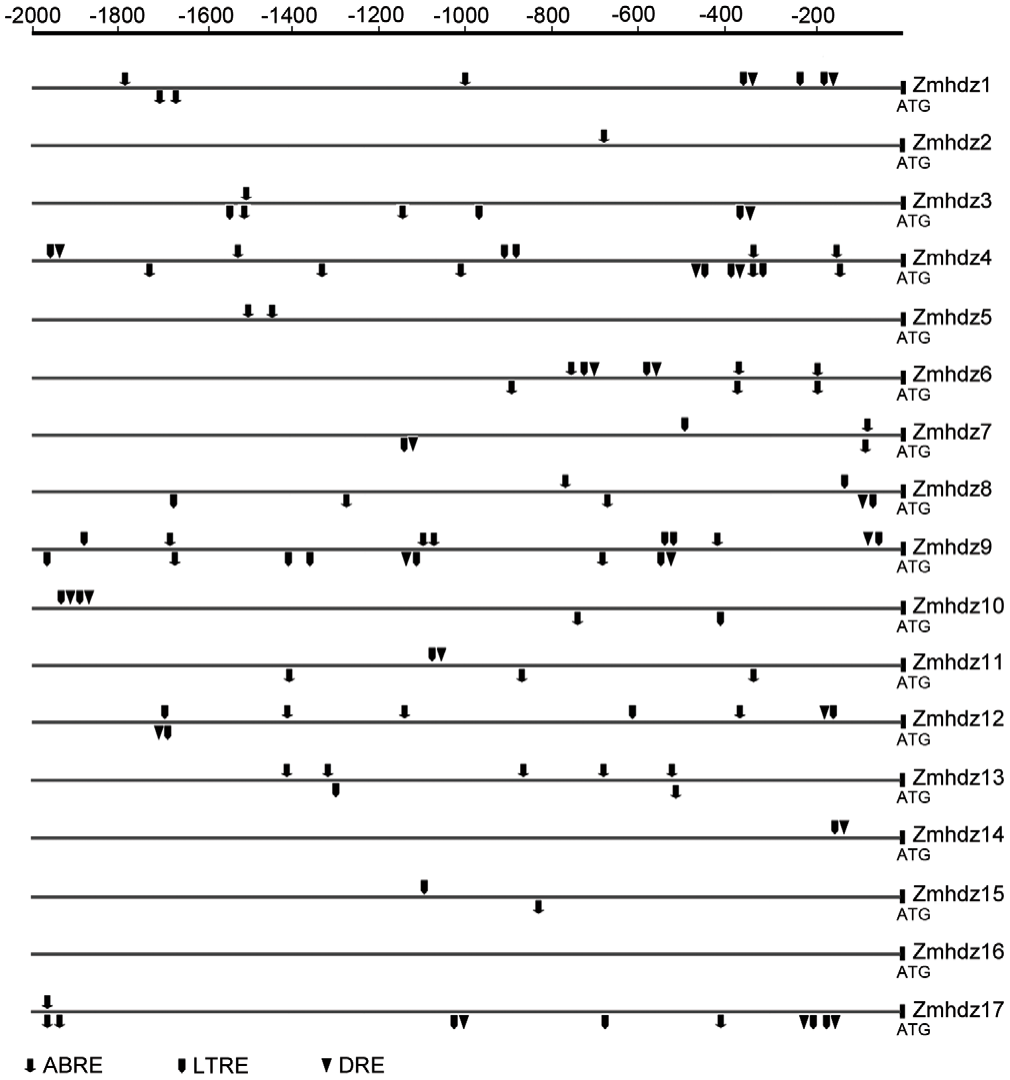
Distribution of major stress-related *cis*-elements in the promoter sequences of the 17 *Zmhdz* I genes. Putative ABRE, LTRE and DRE core sequences are represented by different symbols as indicated. The *cis*-elements distributed on the sense strand and reverse strand are indicated above and below the *black* lines, respectively.

To elucidate the possible regulatory mechanism of *Zmhdz* I genes in various stress responses, we identified putative stress-related *cis*-elements in the 2 kb promoter regions upstream of the transcription start site (ATG) of the stress-responsive *Zmhdz* I genes. Three types of *cis*-elements, which are directly related to the ABA-responsive element (ABRE) [51], [52], low temperature-responsive element (LTRE) [52] and dehydration-responsive element (DRE) [51], were detected by searching the prompter sequence against the PLACE database. Surprisingly, we found that all the *Zmhdz* I genes except for *Zmhdz16* contained the putative ABRE, LTRE or DRE in their promoter regions (Figure 5). Moreover, several members, such as *Zmhdz4*, -*9* and -*12*, were enriched with multiple ABRE, LTRE and DRE in their promoters. By comparing the distribution of the three regulatory elements in the promoter regions, the six sister pairs of HD-Zip I genes were found to exhibit significant differences in their promoter sequences, suggesting that the duplicated genes may not have some regulatory features in common, but rather under similar regulatory pathway in some respects. For example, each of the duplicated genes contained at least one ABRE element in their promoter regions.

### Expression patterns of *Zmhdz* I genes under drought stress

**Figure 6:**
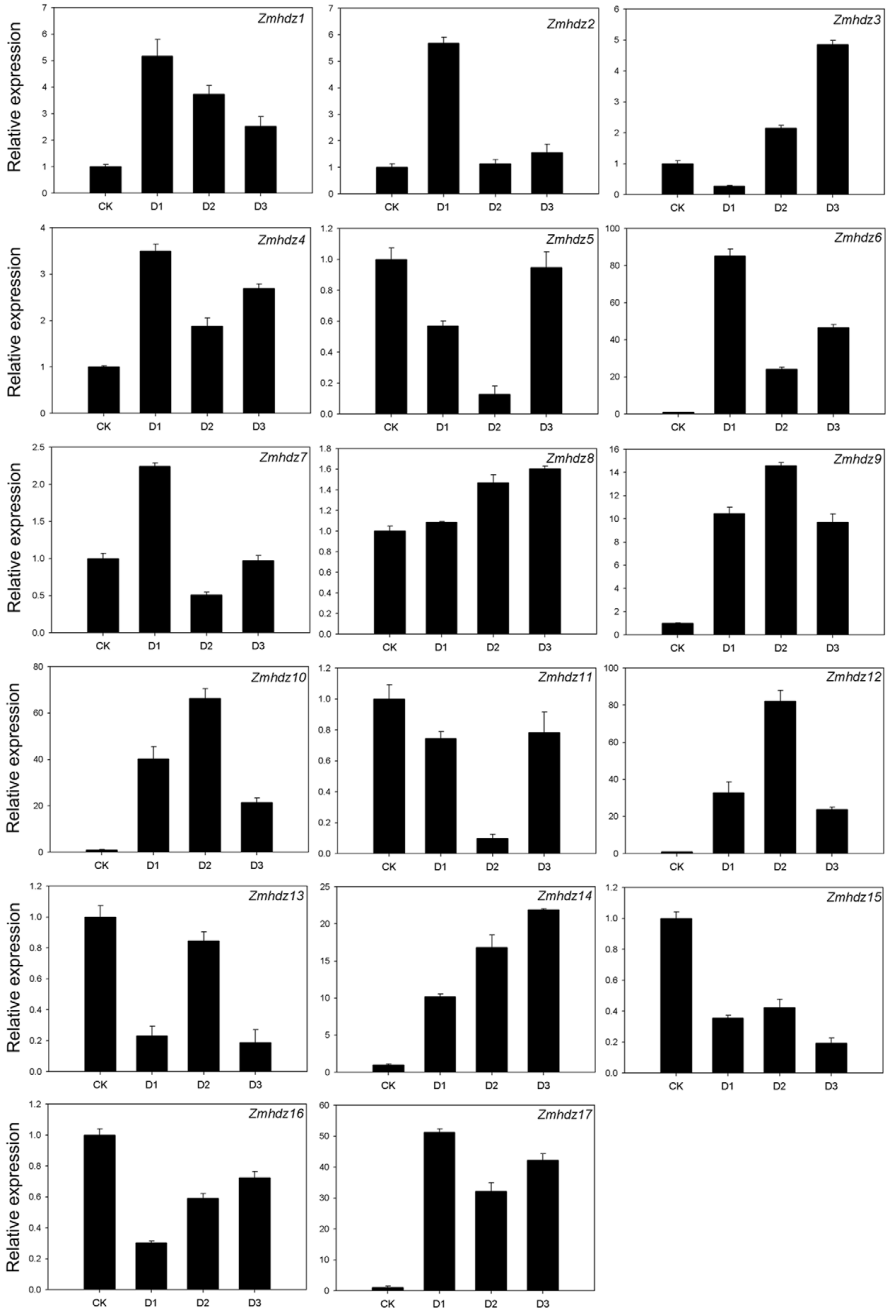
Expression patterns of the 17 *Zmhdz* I genes under drought stress. Relative expressions of *Zmhdz* I genes were examined by qRT-PCR and normalized by *Actin 1* expression. The *Y* axis is the scale of the relative expression level. The *X* axis is the time course of drought stress treatment. *CK*, normal plant, *D1* (slight stress), leaves sampled at 3 h after treatment, *D2* (moderate stress), leaves sampled at 6 h after treatment and *D3* (severe stress), leaves sampled at 12 h after treatment. Date represent mean ± SD.

The HD-Zip I genes have been demonstrated to play important roles in abiotic stress. In this study, we investigated the expression patterns of the *Zmhdz* I genes in three-week-old leaves under drought stress, one of the serious environmental stresses affecting maize production. As shown in figure 6, the expression levels of all the 17 HD-Zip I genes responsive to slight, moderate and severe drought stress were compared with normal plants. Of the 17 HD-Zip I genes, the expressions of 12 *Zmhdz* I genes were obviously up-regulated in response to drought stress, whereas those of the other 5 were down-regulated. It should be noted that 9 out of 12 up-regulated *Zmhdz* I genes showed major expression changes (scales from 0 to 4 up 0 to 100), including *Zmhdz1*, -*2*, -*3*, -*6*, -*9*, -*10*, -*12*, -*14* and -*17*, while the other 3 genes (*Zmhdz4*, -*7* and -*8*) showed minor changes (scales from 0 to 4 and lower). The differential expression patterns were also found among different subclasses based on phylogenetic classification. Drought stress resulted in a down-regulation of *Zmhdz11*, -*5* and -*13* of subclass α. By contrast, the expression levels of the subclass γ genes, including *Zmhdz12*, -*9*, -*6* and -*4*, were strongly induced by more than 2-fold compared to the control. The drought treatment also induced the expressions of *Zmhdz7*, -*1*, -*3* and -*2* of the subclass β1. Interestingly, besides *Zmhdz16*, all genes of the maize-specific ζ clade exhibited significantly up-regulated of their expression levels, especially for *Zmhdz10* and -*17*. In addition, the expression of *Zmhdz15* was greatly down-regulated by drought stress.

The expression profiles of the six duplicated gene pairs were also compared. The results revealed that the duplicated genes within a sister pair exhibited the similar expression patterns upon drought treatment. The different expression patterns between the two duplicated genes were also observed. For example, the highest expression level of *Zmhdz2* was observed at 3 h (D1) after drought treatment, while *Zmhdz3* was observed at 12 h (D3).

A previous study showed up-regulation in expression levels of maize *PSY2* and -*3* under drought stress [53]. Therefore, we investigated the expression pattern of these two genes under drought stress as a control for the drought treatment in this study. Consistent with results of the earlier report, the expression levels of *PSY2* and -*3* were significantly increased under drought conditions (Figure S4A). Furthermore, expression level of *Actin 1*, a traditional housekeeping gene, was compared with a stably expressed gene (*GRMZM2G027378_T01*) reported by Sekhon et al. (2011) under the same drought conditions to examine the reliability of the expression results [54]. As shown in figure S4B, expression of each housekeeping gene did not decrease or in some case increased only to a small extent in the four cDNA samples, suggesting that the expression levels of the two genes were relatively stable under the drought treatment adopted in this study.

## Discussion

### Segmental duplication plays a major role in expansion of the HD-Zip gene family

In the present study, a total of 55 non-redundant HD-Zip genes were identified in the maize genome. However, the number of HD-Zip genes from our search was lower than that previously reported (70) [7]. This difference may be attributed to the fact that the identification of HD-Zip genes in this study was based on the amino acid sequences using BlastP program, whereas the analysis in the previous report was based on the nucleotide sequences using TblastN searches. The *P*-values might affect the number of candidate sequences identified in this step. Moreover, the maize genome database used in our study was the most current with updated sequence assemblies, this may be another reason caused the different number of the HD-Zip genes. Additionally, maize genome contains extensive regions of chromosomal duplication [50]. To exclude potentially redundant sequences from our candidate HD-Zip genes, the criterion used in the two studies could not be completely consistent.

Although the maize genome size is approximately 18 times (2300 Mb: 130 Mb) and the gene number is about 1.3 times (32,000: 25,000) that of *Arabidopsis*, the number of *Zmhdz* genes was found surprisingly to be only seven more than in *Arabidopsis* [8]–[11], [42]. It is believed that tandem and segmental duplications resulted in a substantial expansion in gene family members during the process of genome evolution. Like *Arabidopsis* and rice [55]–[58], the maize genome has undergone several rounds of genome duplication early in its evolution [59], [60]. A total 12 sister pairs of close paralogs were found lying on the segmental duplicated regions among the 55 *Zmhdz* genes, and the other 5 sister pairs were detected as putative segmental duplicated genes. These genes represented approximately 62% of *Zmhdz* genes that have evolved from duplicated chromosomal regions. By contrast, merely five genes (*Zmhdz28*, -*23*, -*22*, -*19* and -*29*) belonging to the HD-Zip II subfamily were found to involve in tandem duplication. Significantly, the number of *Zmhdz* genes involved in segmental duplication was much larger than that those arranged in tandem duplication. On the other hand, the results indicated that segmental duplication was the main contributor to the expansion of maize HD-Zip genes.

At least 75% of *Arabidopsis* HD-Zip genes are involved in segmental duplication, and no obviously clustered HD-Zip genes have been detected on the chromosomes [8]–[11]. The results suggested that the segmental duplication of HD-Zip genes in *Arabidopsis* is more prevalent than in the maize (62%) and rice (54%) genomes [44]. From the results mentioned above, it is clear that segmental duplication was largely responsible for the expansion of HD-Zip gene family in these three species. In general, segmental duplications are thought to occur regularly in more slowly evolving gene families, e.g. MYB gene family [48], [61]. The prevalence of segmental duplication might dominate the slow evolutionary rate of the plant-specific HD-Zip gene family throughout the evolutionary process, resulting in the similar number of HD-Zip genes in the maize, rice and *Arabidopsis* genomes.

### Phylogenetic analysis and evolution of HD-Zip family genes

We have performed a detailed phylogenetic analysis of maize, rice and *Arabidopsis* HD-Zip genes and thus divided *Zmhdz* genes into four distinctly classes. Six monophyletic subclasses of *Arabidopsis* HD-Zip I genes described by Henriksson et al. (2005) were shown in the phylogenetic tree (Figure 2). However, no maize or rice homologs were found in the clades ε and ϕ. The phylogenetic analysis suggested that the six subclasses of HD-Zip I genes share a common origin that may existed in early organisms [8], suggesting that the lineages of clades ε and ϕ genes have been lost in maize and rice [33]. Conversely, an additional clade (ζ) was shown to be specific for maize and rice, implying that it is unique to the grass genomes. A specific subclass of maize and rice class II genes (including *Zmhdz23*, -*28*, -*22*, -*31*, -*24* and *Oshox18* and -*26*) was also identified. The phylogenetic analysis suggested that the genes of this subclass may have diverged from their common ancestral genes predating the monocots and dicots divergence and evolved separately in the grass genomes. Additionally, some branches with low bootstrap values were also found. This phenomenon should be due to the expansion of HD-Zip genes of some clades along with the genome evolution following the monocot-dicot divergence, thus resulting in the sequence divergence of some members. The prevalence of *Zmhdz*-*Oshox* ortholog pairs in the phylogenetic tree indicated that maize HD-Zip genes are more closely allied with rice homologs than *Arabidopsis* representatives, which is consistent with the evolutionary relationships among maize, rice and *Arabidopsis*.

The results of the gene structure analysis not only supported the phylogenetic reconstruction, but also revealed that the HD-Zip genes were highly conserved in evolution. The *Zmhdz* genes within the same class or subclass share similar exon-intron structure, and the domains defined by motif distribution are highly conserved within a class. The conserved evolution of HD-Zip genes was also reflected in the gene duplication. Previous studies revealed that the maize genome experienced several rounds of genome duplication, including an ancient genome duplication that occurred before the divergence of the grass genomes between 50 to 70 million years ago (Mya), resulting in the ancient tetraploid ancestry, an additional whole genome duplication about 5 Mya after the divergence of maize and sorghum and a recent duplication event [42], [50], [62], [63]. Date from the investigation of *Arabidopsis* genome indicated that genome duplication resulted in the expansion of the HD-Zip members was thought to have occurred approximately between 20 to 60 Mya [8], [64], [65]. It is believed that a common ancestor of each clade must have existed prior to the divergence of monocots and dicots, because of all the four clades were found to have representatives from rice, maize and *Arabidopsis* [44]. Thus, pairs of paralogous HD-Zip genes in maize derive from genome duplication were estimated to have taken place in the period of the ancient tetraploid ancestry and the divergence of maize and sorghum. Most orthologs showed a close relationship than paralogs between maize and rice HD-Zip genes, indicating that these genes formed the ortholog pairs might have originated from their common ancestor, in which ancient duplication events occurred predating maize-rice divergence. Those observations combined with the prevalence of segmental duplication of the HD-Zip genes implied that this gene family is a conserved and slowly evolving family in plant genomes.

### Expression analysis of *Zmhdz* I genes in response to drought stress

In plants, quite a few members of the HD-Zip family have been demonstrated to be regulated by dehydration [14], [22], [66]. However, no HD-Zip genes response to drought stress was reported in maize. For this purpose, we investigated the expression patterns of maize HD-Zip I genes under drought stress. The results demonstrated that the expression levels of the 17 *Zmhdz* I genes were either increased or repressed by drought stress. Most genes within the same subclass of the phylogenetic tree showed the similar expression patterns. Since HD-Zip genes in the same subclass share high sequence similarity, the similar expression patterns were also found by comparing the six pairs of duplicated genes, indicating that the regulatory sequences that response to the stress conditions did not diverge much along with the evolution of each gene after duplication and that the duplicated genes might have redundant functions in response to drought stress. It is noteworthy that *Zmhdz I* genes clustered in the maize/rice-specific subclass (ζ clade) were significantly up-regulated under drought stress, with the exception of *Zmhdz16*. *Oshox4*, a rice HD-Zip I gene of the ζ clade, has been demonstrated to play a positive role in response to drought stress [33]. Those findings suggested that genes of the ζ clade are novel to the *Zmhdz* gene family and more likely to play important roles in regulating drought stress. The other five down-regulated genes and three genes with minor expression changes may also have specific functions in maize under drought conditions. A few down-regulated HD-Zip genes have been reported to play positive roles in response to abiotic stress in other plants [32], [33].

In general, duplicated genes have three outcomes: neofunctionalization, subfunctionalization and pseudogenization [67]. Although *Zmhdz16* was not detected as a duplicated gene in the present study, it was closely related to the duplicated genes *Zmhdz14* and -*17*, suggesting that they probably originated from a common ancestral gene. Accordingly, we speculated that the repression of *Zmhdz16* expression under drought stress might indicate its neofunctionalization or subfunctionalization during the course of evolution. Another explanation is that the promoter of *Zmhdz16* does not contain any of the three types of stress-responsive *cis*-elements in the 2 kb promoter region. Some novel stress-responsive *cis*-elements should exist in the promoter region of *Zmhdz16* and play an essential role in regulating stress response. Both hypotheses should be biologically examined in future studies.

## Materials and Methods

### In silico identification and sequence analysis of *Zmhdz* genes

Maize (*Zea mays* L. B73) genome sequences were downloaded from http://www.maizesequence.org/ index.html. The Hidden Markov Model (HMM) profile of the HD domain (PF00046) was obtained from Pfam database (http://pfam.sanger.ac.uk/) [68]. We employed this HMM profile as a query to identify all HD-containing sequences in maize by searching HD domain sequence against the maize genome database using BlastP program (*P*-value  = 0.001). Subsequently, the Pfam and SMART (http://smart.embl-heidelberg.de/) [69] were used to determine each candidate HD protein as a member of the HD-Zip family, which contains both the conserved HD and Zip (PF02183) domains, and this step was crucial to identify the exact number of candidate HD-Zip proteins. Information regarding the number of amino acids and exons, clone number and ORF length of *Zmhdz* genes was obtained from the B73 maize sequence database. The molecular weight (kDa) and isoelectric point (PI) of each gene were calculated by ExPASy (http://www.expasy.org/tools/). All of the candidate HD-Zip sequences were aligned using ClustalW [70] and checked manually to exclude potentially redundant genes, and all of the non-redundant HD-Zip genes were used for further analysis. Analyses of phylogenetic relationships were conducted using MEGA 4.0 [71] with the neighbor-joining (NJ) method. A phylogenetic tree was initially constructed using the complete HD-Zip protein sequences of maize, rice and *Arabidopsis* HD-Zip genes with default parameters [8]–[11], [33]. Bootstrap analysis was performed using 1,000 replicates with the pairwise deletion option. Classification of the *Zmhdz* genes was then performed according to their phylogenetic relationships with their corresponding *Arabidopsis* and rice HD-Zip genes. The unrooted trees were generated using the same method.

The conserved motifs encoded by each *Zmhdz* gene were also investigated. Protein sequences were subjected to online MEME (Multiple Expectation Maximization for Motif Elicitation) (http://meme.sdsc.edu/meme4_3_0/intro.html) [72]. Parameters were set as follows: optimum motif width set to ≥6 and ≤200; maximum number of motifs set to 20. To predict the exon-intron structure of the *Zmhdz* genes, comparison of the genomic sequences and their predicted coding sequences (CDS) was performed using GSDS (http://gsds.cbi.pku.edu.cn/) [73]. The chromosome location image of *Zmhdz* genes was generated by MapInspect (http://www.plantbreeding.wur.nl/ uk/software_mapinspect.html) according to their starting positions in the maize chromosomes. For detection of tandem and segmental duplications, we defined two genes locating in the same clade of the phylogenetic tree as co-paralogs in the same species. Genes were designated as segmental duplication provided that they are co-paralogs and located on duplicated chromosomal blocks as proposed by Wei et al. (2007) [50], [74], [75]. Paralogs were regarded as tandem duplicated genes if two genes were separated by five or fewer genes [76].

Once the starting positions of *Zmhdz* genes were determined on the maize genome, the 2,000 bp genomic sequences upstream of the transcription start site (ATG) were acquired from maize B73 genomic database. PLACE (http://www.dna.affrc.go.jp/PLACE/signalscan.html), an online database of plant *cis*-acting regulatory DNA elements (*cis*-elements) [77], was employed to investigate putative *cis*-elements in the promoter regions of the *Zmhdz* genes.

### Plant materials and drought stress treatment

In this study, three-week-old seedlings (five-leaf stage) of maize inbred line B73 were used to examine the expression patterns of HD-Zip I genes under drought stress. Plants were grown in a greenhouse at 28±2°C with a 14-h light/10-h dark photoperiod. For drought stress, three-week-old plants were treated by putting intact plants in air at 28±2°C with water-limiting conditions, and plant leaves of the seedlings were harvested at 0, 3, 6 and 12 h after drought treatment, which represented normal plants, slight stress, moderate stress and severe stress, respectively. Three replicates were conducted for each sample.

### RNA isolation and quantitative real-time PCR (qRT-PCR)

Total RNAs were isolated from the collected samples using Trizol reagent (Invitrogen) and treated with DNase I (Invitrogen) for 20 min to remove possible contaminating genomic DNA. Total RNA was assessed on a 1.2% agarose gel and quantified by NanoDrop ND-1000 spectrophotometer. For qRT-PCR analysis, first-strand cDNAs were synthesized from 1 µg of total RNA using Superscript II reverse transcriptase (Invitrogen) according to the manufacturer's instructions. Real-time PCR was performed on an ABI 7300 Real-Time system (Applied Biosystems). Each reaction was done in a final volume of 25 µl containing 12.5 µl of SYBR Green Master Mix reagent (Applied Biosystems), 2.0 µl of cDNA sample, and 400 nM gene-specific primers. The specific primers of each *Zmhdz* I gene (Table S2) were designed to generate a size of 90–150 bp products using Primer Express 3.0 software (Applied Biosystems). The thermal cycle conditions were as follows: 50°C for 2 min, 95°C for 10 min, 40 cycles of 95°C for 15 s and 60°C for 1 min. At the end of the 40 cycles, a melting curve was generated to analyze the specificity of the reactions. Each cDNA sample was tested in three replicates. Expression level of the maize *Actin 1* gene was used as the endogenous control. The relative expression level was calculated as 2^–ΔΔ*C*T^ [Δ*C*_T_  =  *C*_T, gene of interest_ − *C*_T, *Actin 1*_. ΔΔ*C*_T_  =  Δ*C*_T, drought treatment_ − Δ*C*_T, CK (0h)_]. The relative expression level (2^–ΔΔ*C*T, CK (0h)^) in the normal plants without drought treatment was normalized to 1 as described previously [78]. Statistical analyses were conducted using the SDS software 1.3.1 (Applied Biosystems).

## Supporting Information

Figure S1 **MP phylogeny of maize, rice and** ***Arabidopsis*** **HD-Zip proteins.** The MP tree was generated with MEGA5.0 program using the full-length amino acid sequences of the maize, *Arabidopsis* and rice HD-Zip proteins. The bootstrap analysis was performed using 1,000 replicates with the pairwise deletion option. The tree was divided into four classes, and was largely consistent with the results of the NJ tree in figure 2.
(TIF)

Figure S2 **ML phylogeny of maize, rice and** ***Arabidopsis*** **HD-Zip proteins.** The ML tree was generated with MEGA5.0 program using the full-length amino acid sequences of the maize, *Arabidopsis* and rice HD-Zip proteins. The bootstrap analysis was performed using 1,000 replicates with the pairwise deletion option. The tree was classified into four classes, and was largely consistent with the results of the NJ tree in figure 2.
(TIF)

Figure S3 **Multiple sequence alignment of 55 maize HD-Zip proteins.** The complete amino acid sequences of the 55 *Zmhdz* proteins were aligned using ClustalW and revised manually. The HD, Zip, START and MEKHLA domains are indicated by white box, grey box, straight line and dashed line, respectively. Due to the diverse structures of class III and IV proteins, the conserved domains were cycled by black rectangles.
(TIF)

Figure S4 **Analysis of the expression patterns of known drought-responsive and housekeeping genes by qRT-PCR.** (A) Expression levels of two drought-responsive genes in the maize B73 seedlings at the five-leaf stage were analyzed under drought stress. (B) Expression levels of the *Actin 1* gene and one putative housekeeping gene were compared under the same drought treatment.
(TIF)

Table S1 **Sequence characteristics of 55 HD-Zip genes identified in maize.**
(DOC)

Table S2 **Specific primers used for qRT-PCR in this study.** Primer sequences used for qRT-PCR to validate the expression patterns of *Zmhdz* I genes under drought stress.
(XLS)

Text S1 **Amino acid sequences of maize, rice and** ***Arabidopsis*** **HD-Zip proteins used for phylogenetic analysis.**
(TXT)
