# Supplementary figures and images for: Systematic Analysis of Sequences and Expression Patterns of Drought-Responsive Members of the HD-Zip Gene Family in Maize
Source: PLoS One. 2011 Dec 2;6(12):e28488. doi: 10.1371/journal.pone.0028488 (PMC3229603; doi:10.1371/journal.pone.0028488)

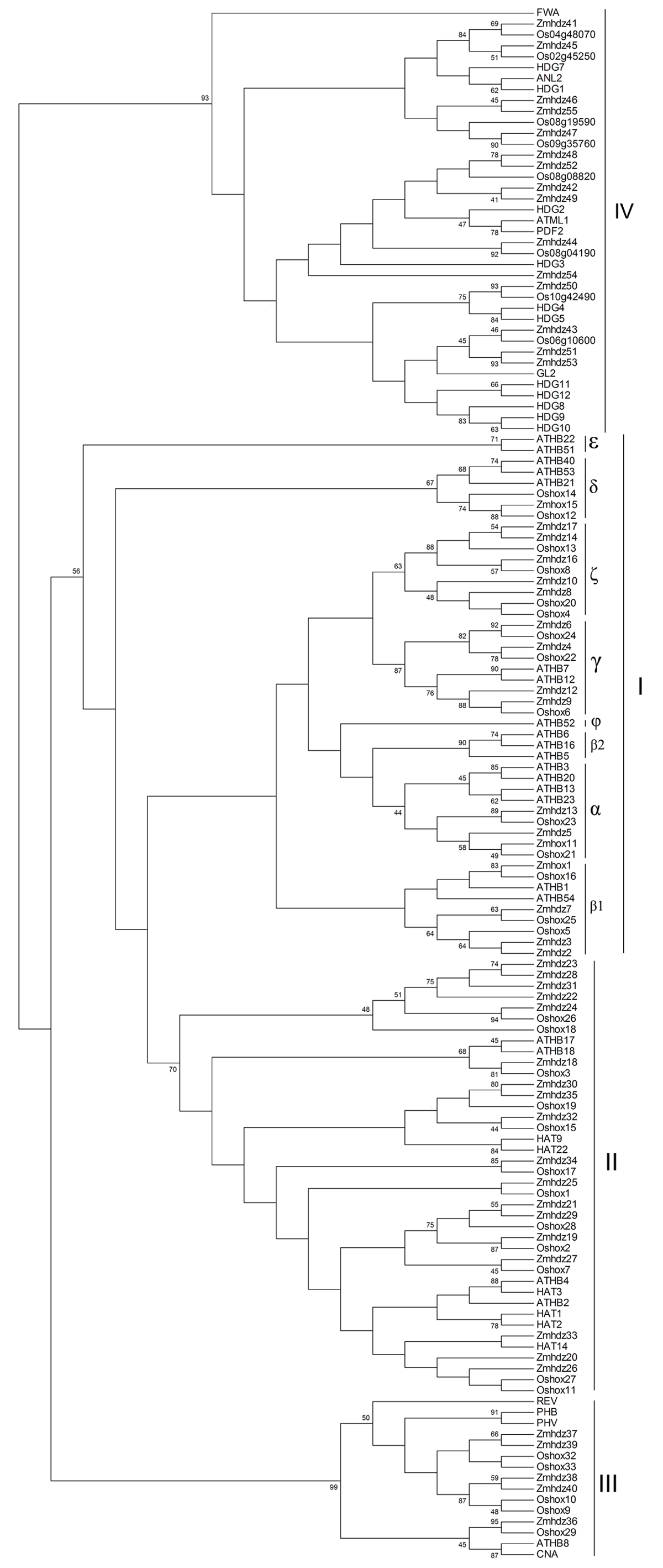

Supplement: Figure S1 — MP phylogeny of maize, rice and Arabidopsis HD-Zip proteins. The MP tree was generated with MEGA5.0 program using the full-length amino acid sequences of the maize, Arabidopsis and rice HD-Zip proteins. The bootstrap analysis was performed using 1,000 replicates with the pairwise deletion option. The tree was divided into four classes, and was largely consistent with the results of the NJ tree in figure 2. (TIF) [file pone.0028488.s001.tif]

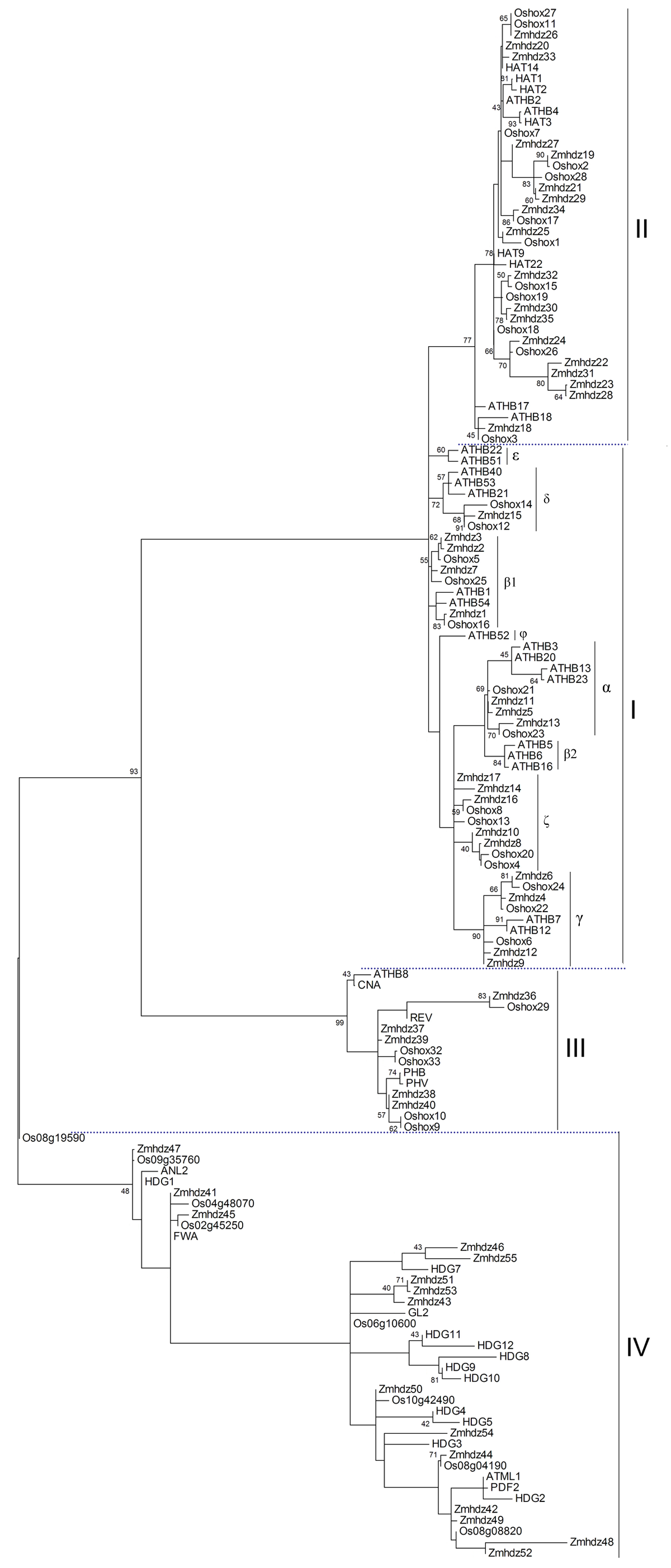

Supplement: Figure S2 — ML phylogeny of maize, rice and Arabidopsis HD-Zip proteins. The ML tree was generated with MEGA5.0 program using the full-length amino acid sequences of the maize, Arabidopsis and rice HD-Zip proteins. The bootstrap analysis was performed using 1,000 replicates with the pairwise deletion option. The tree was classified into four classes, and was largely consistent with the results of the NJ tree in figure 2. (TIF) [file pone.0028488.s002.tif]

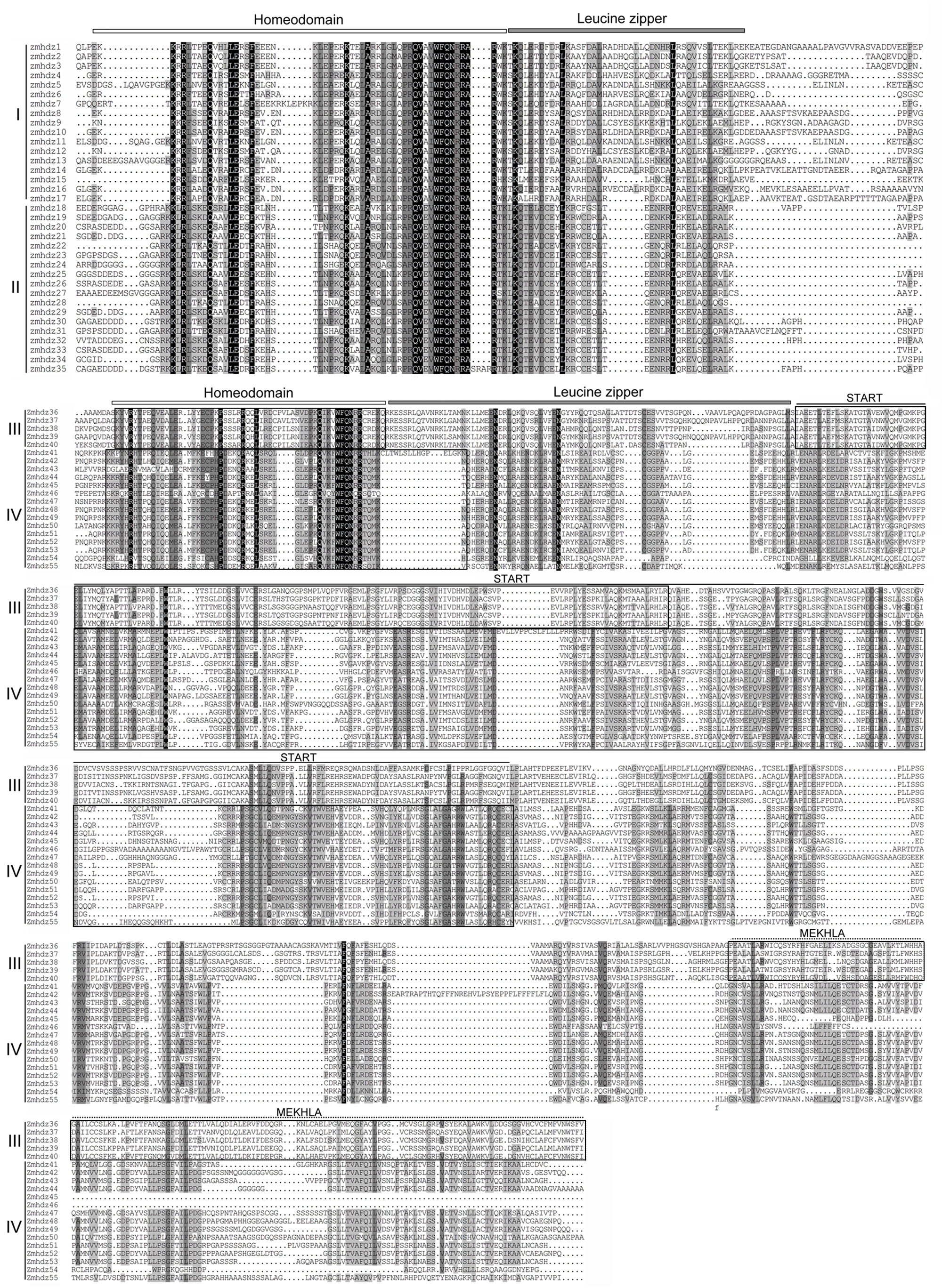

Supplement: Figure S3 — Multiple sequence alignment of 55 maize HD-Zip proteins. The complete amino acid sequences of the 55 Zmhdz proteins were aligned using ClustalW and revised manually. The HD, Zip, START and MEKHLA domains are indicated by white box, grey box, straight line and dashed line, respectively. Due to the diverse structures of class III and IV proteins, the conserved domains were cycled by black rectangles. (TIF) [file pone.0028488.s003.tif]
