# Supplementary material for: Systematic Analysis of Sequences and Expression Patterns of Drought-Responsive Members of the HD-Zip Gene Family in Maize
Source: PLoS One. 2011 Dec 2;6(12):e28488. doi: 10.1371/journal.pone.0028488 (PMC3229603; doi:10.1371/journal.pone.0028488)
Supplement: Table S1 — Sequence characteristics of 55 HD-Zip genes identified in maize. (DOC) [file pone.0028488.s005.doc]

**Table S1.** **Sequence characteristics of 55 HD-Zip genes identified in maize.**

| Gene  code ^a^ | Accession Number | | Chr.^b^ | Protein | | | ORF  (bp) | Exons | Type |
| --- | --- | --- | --- | --- | --- | --- | --- | --- | --- |
|  | Ensembl transcript ID | Clone no. |  | Length  (aa) | MW  (kDa) | PI |  |  |  |
| Zmhdz1  Zmhdz2  Zmhdz3  Zmhdz4  Zmhdz5  Zmhdz6  Zmhdz7  Zmhdz8  Zmhdz9  Zmhdz10  Zmhdz11  Zmhdz12  Zmhdz13  Zmhdz14  Zmhdz15  Zmhdz16  Zmhdz17  Zmhdz18  Zmhdz19  Zmhdz20  Zmhdz21  Zmhdz22  Zmhdz23  Zmhdz24  Zmhdz25  Zmhdz26  Zmhdz27  Zmhdz28  Zmhdz29  Zmhdz30  Zmhdz31  Zmhdz32  Zmhdz33  Zmhdz34  Zmhdz35  Zmhdz36  Zmhdz37  Zmhdz38  Zmhdz39  Zmhdz40  Zmhdz41  Zmhdz42  Zmhdz43  Zmhdz44  Zmhdz45  Zmhdz46  Zmhdz47  Zmhdz48  Zmhdz49  Zmhdz50  Zmhdz51  Zmhdz52  Zmhdz53  Zmhdz54  Zmhdz55 | GRMZM2G021339_P01  GRMZM2G122076_P01  GRMZM2G003304_P01  GRMZM2G351330_P01  GRMZM2G178741_P02  GRMZM2G117164_P01  GRMZM2G002915_P01  GRMZM2G056600_P01  GRMZM2G041462_P01  GRMZM2G041127_P03  GRMZM2G139963_P01  GRMZM2G034113_P01  GRMZM2G097349_P01  GRMZM2G132367_P01  GRMZM2G005624_P01  GRMZM2G119999_P01  AC233899.1_FGP004  GRMZM2G126239_P01  GRMZM2G134260_P01  GRMZM2G366130_P01  GRMZM2G068672_P01  GRMZM2G307400_P01  GRMZM2G307397_P01  GRMZM2G166041_P01  GRMZM2G148074_P01  GRMZM2G142962_P02  GRMZM2G047715_P01  GRMZM2G477415_P01  GRMZM2G106276_P01  GRMZM2G105834_P01  GRMZM2G478937_P01  GRMZM2G131476_P01  GRMZM2G127537_P01  GRMZM2G044752_P01  GRMZM2G126808_P02  AC187157.4_FGP005  GRMZM2G003509_P01  GRMZM2G109987_P02  GRMZM2G178102_P01  GRMZM2G469551_P01  AC235534.1_FGP007  GRMZM2G001289_P01  GRMZM2G004334_P01  GRMZM2G004957_P02  GRMZM2G026643_P01  GRMZM2G109252_P01  GRMZM2G116658_P01  GRMZM2G118063_P01  GRMZM2G122897_P01  GRMZM2G123140_P01  GRMZM2G126646_P01  GRMZM2G130442_P01  GRMZM2G145690_P01  GRMZM2G386276_P01  GRMZM2G438260_P01 | AC187915.3  AC185262.2  AC214476.2  AC206625.3  AC191092.3  AC196575.3  AC194663.3  AC206905.3  AC193647.3  AC201765.4  AC211214.3  AC195803.2  AC199175.2  AC177946.2  AC195802.3  AC194676.3  AC233899.1  AC214372.3  AC208890.3  AC203051.3  AC203320.3  AC196796.4  AC196796.4  AC191405.3  AC205663.2  AC203363.3  AC205281.3  AC196796.4  AC208890.3  AC203377.3  AC206319.2  AC207190.3  AC199906.3  AC200743.3  AC197594.3  AC187157.4  AC186423.3  AC204385.3  AC188824.2  AC209904.3  AC235534.1  AC177898.2  AC210830.3  AC188829.3  AC196029.2  AC202082.2  AC191037.3  AC189883.3  AC183312.5  AC205513.3  AC194980.3  AC211472.3  AC199005.2  AC204626.3  AC210174.3 | 4  4  1  2  9  5  2  7  7  2  1  2  1  1  1  1  9  3  9  2  6  5  5  4  1  1  4  5  9  9  6  1  7  2  1  8  1  9  3  1  10  2  6  10  3  7  7  10  10  1  4  4  5  3  2 | 339  272  270  261  344  235  283  261  239  274  344  244  363  326  239  294  377  259  293  315  261  223  222  269  319  448  346  221  272  296  303  262  333  227  296  858  854  842  953  835  863  830  732  769  702  611  863  802  742  884  698  796  692  701  750 | 36.99  30.04  29.99  29.38  37.57  26.41  31.22  28.46  26.24  29.64  37.60  26.79  38.89  35.53  26.54  32.25  40.64  28.61  31.10  33.49  27.37  24.52  24.26  28.27  34.57  46.75  37.14  23.80  28.03  31.56  34.33  28.24  36.02  25.05  31.43  92.03  92.57  92.38  103.56  91.70  92.99  89.65  80.14  83.01  75.61  66.45  91.54  86.06  79.52  94.36  76.17  85.61  75.81  76.97  82.77 | 4.62  4.79  5.00  5.00  6.04  5.01  5.34  4.76  5.39  4.61  6.28  5.38  5.74  5.13  6.21  4.82  7.53  9.17  9.37  8.50  9.50  9.71  9.06  9.65  8.69  9.06  5.39  9.20  9.45  9.21  9.18  8.43  6.98  9.05  9.14  5.72  6.54  5.94  8.29  5.83  6.32  5.67  5.90  5.92  5.51  6.52  5.88  5.43  5.38  5.43  6.19  5.53  6.15  8.20  5.58 | 1020  819  813  786  1035  708  852  786  720  825  1035  735  1092  981  720  885  1134  780  882  948  786  672  669  810  960  1347  1041  666  819  891  912  789  1002  684  891  2577  2565  2529  2862  2508  2592  2493  2199  2310  2109  1836  2592  2409  2229  2655  2097  2391  2079  2106  2253 | 4  3  3  2  3  2  3  3  2  2  3  2  2  3  3  3  3  4  2  3  2  3  4  2  4  4  4  3  2  3  7  3  4  4  3  17  18  18  18  18  9  9  9  6  9  7  9  10  8  10  10  10  10  10  8 | I  I  I  I  I  I  I  I  I  I  I  I  I  I  I  I  I  II  II  II  II  II  II  II  II  II  II  II  II  II  II  II  II  II  II  III  III  III  III  III  IV  IV  IV  IV  IV  IV  IV  IV  IV  IV  IV  IV  IV  IV  IV |

^a^ Name assigned to maize HD-Zip genes in this study.

^b^ Chromosomal localization of the *Zmhdz* genes.
